# Recent Advances of Deep Learning for Computational Histopathology: Principles and Applications

**DOI:** 10.3390/cancers14051199

**Published:** 2022-02-25

**Authors:** Yawen Wu, Michael Cheng, Shuo Huang, Zongxiang Pei, Yingli Zuo, Jianxin Liu, Kai Yang, Qi Zhu, Jie Zhang, Honghai Hong, Daoqiang Zhang, Kun Huang, Liang Cheng, Wei Shao

**Affiliations:** 1 MIIT Key Laboratory of Pattern Analysis and Machine Intelligence, College of Computer Science and Technology, Nanjing University of Aeronautics and Astronautics, Nanjing 211106, China; sx2116019@nuaa.edu.cn (Y.W.); huangshuo@nuaa.edu.cn (S.H.); zongxiang.pei@nuaa.edu.cn (Z.P.); yinglizuo@nuaa.edu.cn (Y.Z.); liujianxin@nuaa.edu.cn (J.L.); yangkai_ibrain@nuaa.edu.cn (K.Y.); zhuqi@nuaa.edu.cn (Q.Z.); dqzhang@nuaa.edu.cn (D.Z.); 2 Department of Medicine, Indiana University School of Medicine, Indianapolis, IN 46202, USA; miccheng@iu.edu (M.C.); jizhan@iu.edu (J.Z.); kunhuang@iu.edu (K.H.); 3 Regenstrief Institute, Indiana University, Indianapolis, IN 46202, USA; 4 Department of Clinical Laboratory, The Third Affiliated Hospital of Guangzhou Medical University, Guangzhou 510006, China; honghonghai@gzhmu.edu.cn; 5 Departments of Pathology and Laboratory Medicine, Indiana University School of Medicine, Indianapolis, IN 46202, USA

**Keywords:** artificial intelligence, machine learning, digital pathology image analysis, color normalization, segmentation, diagnosis and prognosis, a whole-slide pathological imaging (WSI)

## Abstract

**Simple Summary:**

The histopathological image is widely considered as the gold standard for the diagnosis and prognosis of human cancers. Recently, deep learning technology has been extremely successful in the field of computer vision, which has also boosted considerable interest in digital pathology analysis. The aim of our paper is to provide a comprehensive and up-to-date review of the deep learning methods for digital H&E-stained pathology image analysis, including color normalization, nuclei/tissue segmentation, and cancer diagnosis and prognosis. The experimental results of the existing studies demonstrated that deep learning is a promising tool to assist clinicians in the clinical management of human cancers.

**Abstract:**

With the remarkable success of digital histopathology, we have witnessed a rapid expansion of the use of computational methods for the analysis of digital pathology and biopsy image patches. However, the unprecedented scale and heterogeneous patterns of histopathological images have presented critical computational bottlenecks requiring new computational histopathology tools. Recently, deep learning technology has been extremely successful in the field of computer vision, which has also boosted considerable interest in digital pathology applications. Deep learning and its extensions have opened several avenues to tackle many challenging histopathological image analysis problems including color normalization, image segmentation, and the diagnosis/prognosis of human cancers. In this paper, we provide a comprehensive up-to-date review of the deep learning methods for digital H&E-stained pathology image analysis. Specifically, we first describe recent literature that uses deep learning for color normalization, which is one essential research direction for H&E-stained histopathological image analysis. Followed by the discussion of color normalization, we review applications of the deep learning method for various H&E-stained image analysis tasks such as nuclei and tissue segmentation. We also summarize several key clinical studies that use deep learning for the diagnosis and prognosis of human cancers from H&E-stained histopathological images. Finally, online resources and open research problems on pathological image analysis are also provided in this review for the convenience of researchers who are interested in this exciting field.

## 1. Introduction

**Figure 1:**
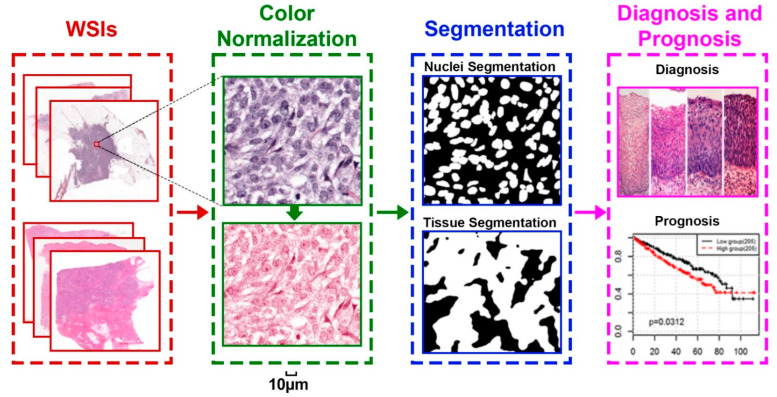
General research directions for digital pathology image analysis.

Cancer is the second leading cause of mortality worldwide. It is reported that the global cancer burden is expected to be 28.4 million cases in 2040 [1]. Thus, the effective and efficient diagnosis of human cancer, especially at its early stage, is essential for global cancer control. Recently, a wide variety of biomarkers have been utilized for the diagnosis and prognosis of cancers, including radiomics images [2], histopathological images, and genetic signatures, such as genetic mutations, gene expression, and protein markers [3]. Among these, the histopathology image is widely recognized as the “golden standard” for analyzing human cancers since it can visually reflect the aggressiveness of human cancers at the cell level [4]. Recently, with the remarkable success of digital histopathology, whole-slide imaging (WSI) has become more advanced and has been frequently used for the diagnosis and prognosis of human cancers, since it excels at characterizing the morphology within the tissue at high resolution [5]. Hematoxylin and eosin (H&E) staining is the most commonly used tissue staining method in the world. Generally, the research directions for the analysis of H&E-stained WSI can be summarized into the components of color normalization, segmentation, and cancer diagnosis/prognosis (shown in Figure 1). Specifically, color normalization is used to preprocess the images to correct staining variations across different images. WSI segmentation is used to segment the nuclei or tissues from the WSI. Finally, the prediction models are designed for the diagnosis and prognosis of human cancers. However, due to the time-consuming inspection of WSI and the large inter-operator variation among pathologists, there is an imperative need to develop machine learning models to automatically analyze H&E-stained histopathological images in a more reliable way [6].

The machine learning-based methods for the analysis of H&E-stained histopathological images can be divided into two categories (i.e., the traditional machine learning methods and deep learning methods). The traditional computational methods objectively evaluate disease-related tissue changes by extracting handcrafted features such as textural [7] and morphological features [8], followed by designing classifiers such as support vector machine (SVM) [9], random forest (RF) [10] and K-nearest neighbors (K-NN) [11] for the downstream analysis tasks. For instance, Kruk et al. [12] first extracted morphometric, textural, and statistical features from the WSI, and then used these features for nuclei classification by the combination of SVM and RF classifiers. Fuchs et al. [13] proposed a computational pipeline to extract local binary patterns and color features from images and then used these features to segment nuclei relying on a RF classification model. Zeralla et al. [14] firstly extract the spatial feature from WSI, then the SVM classifier is applied to accomplish the color normalization task. It has been proved that traditional machine learning algorithms could achieve significantly superior classification performance than their competitors if the sample size for model training is small [15], which is suitable for analyzing rare cancer subtypes with a limited sample size [16]. Moreover, traditional machine learning models are more understandable and explainable, and can be used to help clinicians understand how the machine learning models make decisions.

Although much progress has been made, three common limitations have existed in the traditional machine learning methods for H&E-stained histopathological image analysis. First, the handcrafted features are extracted in an unsupervised way and are uncorrelated with the following WSI analysis task [17]. Secondly, the extracted handcrafted features can only learn the shallow representation of the input image, given the heterogenous patterns of WSI, these shallow model-based feature extraction methods may be insufficient to characterize the complex WSI [18]. Thirdly, most traditional machine learning algorithms are designed for data that would be completely loaded into memory, which is difficult for analyzing large amounts of WSI [19]. Recently, deep learning technology has been extremely successful in the field of computer vision, which also boosts considerable interest in digital H&E-stained pathology analysis [20,21,22]. In comparison with traditional machine learning approaches, the deep learning algorithms go directly from the input to the desired output to extract useful features for specific WSI analysis tasks, which can avoid the complex feature extraction step. In addition, the heterogenous patterns of WSI can cause variance across different samples, thereby causing the difficulties of handcrafted features with limited generalization abilities [23]. The deep learning algorithms are capable of characterizing such complex patterns when given amounts of WSI data for model training. Moreover, given recent advances in the high-throughput tissue bank and archiving of digitized WSI, the deep learning algorithms are much more scalable due to their ability to process massive amounts of data and perform a lot of computations in a cost and time-effective manner [24].

In this paper, we systematically review the research directions and challenges of deep learning methods for H&E-stained histopathological image analysis (shown in Figure 1). Our paper is organized as follows. In Section 2, we will briefly introduce the concepts and structure of the deep neural network. In Section 3, we will introduce the research direction of color normalization for the H&E-stained histopathological image analysis. In Section 4, we will summarize the literatures that applied the deep learning method for various H&E image segmentation tasks such as nuclei and tissue segmentation. In Section 5, we will review the clinical studies that apply H&E-stained histopathological images for the diagnosis and prognosis of cancer based on H&E-stained histopathological images. Finally, online resources and open research problems on H&E-stained histopathology image analysis are also provided in Section 6.

## 2. Deep Neural Network

Deep learning is a new research direction in the field of machine learning based on the deep neural network, which has greatly boosted the performance of natural image analysis techniques, such as image classification [24], object detection [25], and semantic segmentation [26].

**Figure 2:**
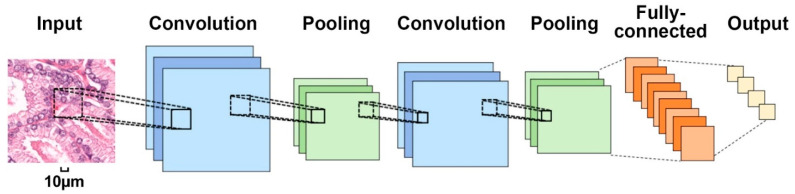
The general architecture of a convolutional neural network.

A deep neural network is composed of multiple nonlinear modules which can be regarded as a feature learning process from low to high levels. The convolutional neural network (CNN) is the most widely used artificial neural network [27] (shown in Figure 2), which can be regarded as a feature learning process from low to high level. Specifically, the convolutional layers are used to learn local features (i.e., corners and edges from the images). Different convolutional layers are interleaved with the pooling layers, which are used to reduce the output from the convolutional layers. The last fully connected layers are used to combine the features, which are learned from the convolutional layers together and by which we can obtain complex and high-level representation for the final prediction task. We compare and summarize typical CNN (i.e., AlexNet [28], ZFNet [29], VGGNet [30], GoogLeNet [31], ResNet [32], and SENet [33]) from the perspectives of network structure, calculation speed, and classification performance in Table 1, where the additional dropout layer is used to reduce the risk of overfitting [28], while the batch normalization strategy [32] can help diminish the reliance of gradients on the scale of the parameters or their underlying values. CNN takes raw images (or large patches) as input to avoid the complex feature extraction step, which is highly invariant to translation, scaling, inclination, and other forms of deformation. Histopathology images are characterized by data complexity, making deep learning algorithms extremely suitable for each step in pathological image analysis, including color normalization, histopathological image segmentation, and the diagnosis and prognosis of human cancers. We will review them in the following sections.

## 3. Color Normalization

**Figure 3:**
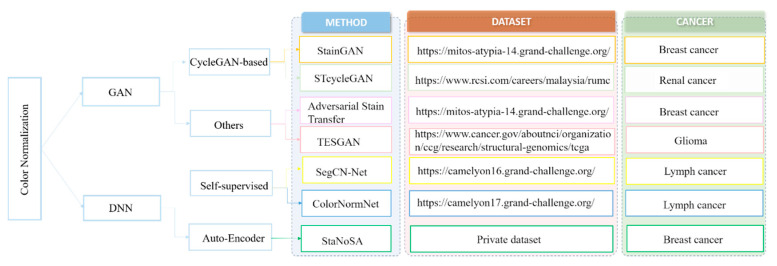
Overview of papers using deep learning for color normalization in histopathological images [35,36,37,38,39,40,41].

Color variations usually exist in WSI due to differences in raw materials and staining protocols across different pathology labs, interpatient variabilities, and slide scanner variations. Intuitively, such color variance will affect the generalization performance of deep learning models. Normalization of the color represented by WSI is thus an important preprocessing task for digital pathology analysis [34]. Herein, we discuss literature on the use of deep learning-based methods for color normalization in histopathological images (Figure 3).

In general, traditional color normalization methods (i.e., color matching and stain separation [42,43,44]) mainly rely on the predefined template image and cannot conduct the style transformation between different image datasets. In principle, this style transformation can be resolved by the deep learning-based methods due to their complicated network structure [39,40,45]. For instance, Patli et al. [40] proposed a self-supervised, learning-based lightweight neural network to estimate the color shift from the source stain to a predetermined target stain in appearance. Bug et al. [45] used a pre-trained deep neural network as a feature extractor steering a pixel-wise normalization pipeline, which can achieve excellent normalization results and ensure a consistent representation of color and texture. Janowczyk et al. [41] presented a novel stain normalization algorithm based on sparse autoencoder (StaNoSa) to standardize the color distribution of input images. The results indicated that StaNoSa showed either comparable or superior results to its competitors.

**Figure 4:**
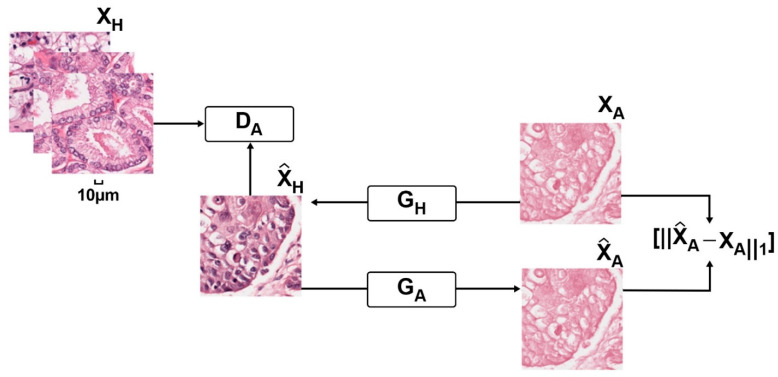
Stain style transfer for digital histopathological images.

Recently, with the rapid development of deep learning, generative adversarial network (GAN) [36] is also widely used to normalize the patches without the guidance of the template images but can still preserve the organization structure of the tissues. For example, BenTaieb et al. [37] designed a discriminative image analysis model equipped with the GAN component that transferred stains across datasets. However, its performance was largely determined by the auxiliary tasks requiring extra labeling efforts. In order to reduce the labeling efforts for experts, Zanjani et al. [46] proposed a novel unsupervised generative model, which was trained in an end-to-end manner and could be instantly applied to unseen images. Inspired by the cycle-GAN [47], which could be successfully applied to image-style transformation, Shaban et al. [35] proposed a framework named StainGAN, which could achieve better qualitative performance in normalizing different images (Figure 4). In addition, other works [38,39] also considered the structural integrity of the histopathological images and integrated semantic information at different layers between a pre-trained semantic network and the stain color normalization network to further improve the normalization performance.

## 4. Pathology Image Segmentation

The segmentation task, which aims at assigning a class label to each pixel of an image, is a common task in pathology image analysis [48]. The segmentation task on histopathological images can be divided into two categories, nuclei segmentation, and tissue segmentation. The nuclei segmentation task focuses on exploring the nuclei features, such as morphological appearances in histopathological images, which are widely recognized as the most frequently used biomarkers for cancer histology diagnosis. On the other hand, the tissue segmentation task takes the histopathology image as input and segments the tissues that are composed of a group of cells in the input image with certain characteristics and structures (i.e., gland, tumor-infiltrating lymphocytes, etc.). These quantitatively measured tissues are also a crucial indicator for the diagnosis and prognosis of human cancers [49,50].

Due to the heterogenous patterns in WSI, the accurate segmentation of nuclei and tissues in the histopathological images is with huge challenges. First, there are variations on nucleus/tissue sizes and shape, requiring a segmentation model with a strong generalization ability. Second, nuclei/cells are often clustered into clumps so that they might partially overlap or touch one another, which will lead to the under-segmentation of histopathological images. Third, in some malignant cases, such as moderately and poorly differentially adenocarcinomas, the structure of the tissues (such as the glands) are heavily degenerated, making them difficult to discriminate [51,52].

**Figure 5:**
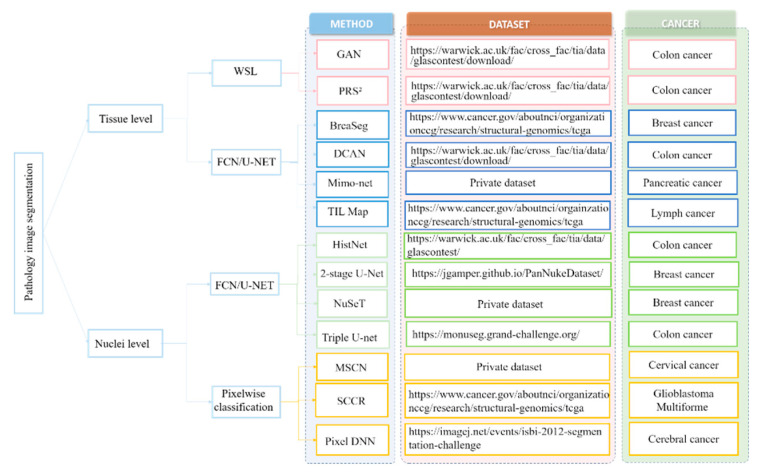
Overview of papers using deep learning for nuclei/tissue segmentation in histopathological images [51,53,54,55,56,57,58,59,60,61,62,63,64].

In view of these challenges, numerous deep learning-based approaches have been proposed to extract high-level features from WSI that can achieve enhanced segmentation performance. Here, we first review the deep learning-based nuclei segmentation algorithm. Then, we summarize the development of deep learning algorithms on tissue-level segmentation tasks. We show the overview of papers using deep learning for nuclei/tissue segmentation in Figure 5.

### 4.1. Nuclei-Level Segmentation

Cellular object segmentation is a prerequisite step for the assessment of human cancers [65]. For example, the counting of mitoses is one of the most prognostic factors in breast cancer requiring the assistance of nuclei segmentation [66]. In the diagnosis of cervical cytology, nuclei segmentation is necessary to discover all types of cytological abnormalities [67]. The traditional nuclei segmentation algorithms are based on morphological processing methods [8], clustering algorithms [68], level set methods [69], and their variants [70,71,72], whose performance are largely determined by the designed features requiring the domain knowledge of experts. Recently, deep learning approaches have been widely applied without the efforts of designing hand-crafted features [73].

Generally, the deep learning-based nuclei segmentation algorithms can be divided into two categories, the pixel-wise classification methods [64,74,75,76] and the fully convolutional network (FCN)-based methods [60,61,77]. Pixel-wise classification methods convert the segmentation task into the classification task, by which the label of each pixel is predicted from raw pixel values in a square window centered on it [74]. For example, Cireşan et al. [64] first densely sampled the squared windows from the WSI, followed by classifying the centered pixels via utilizing the rich context information within the sampled windows. Moreover, Zhou et al. [63] learned a bank of convolutional filters and a sparse linear regressor to produce the likelihood for each pixel being nuclear or background regions. By considering the windows of different sizes can extract helpful complementary information for the nuclei segmentation, a multiscale convolutional network and graph-partitioning–based method [62] were proposed for the task of nuclei segmentation. In addition, Xing et al. [78] firstly learned a CNN model to generate a probability map of each image. According to the probability map, each pixel is then assigned a probability belonging to the nucleus. Finally, an iterative region merging algorithm was used to accomplish the segmentation task. Nesma et al. [79] also presented an optimized pixel-based classification model by the cooperation of region growing strategy that could successfully obtain nucleus and cytoplasm segmentation results. Additionally, Liu et al. [75] proposed a panoptic segmentation model which incorporates an auxiliary semantic segmentation branch with the instance branch to integrate global and local features for nuclei segmentation.

**Figure 6:**
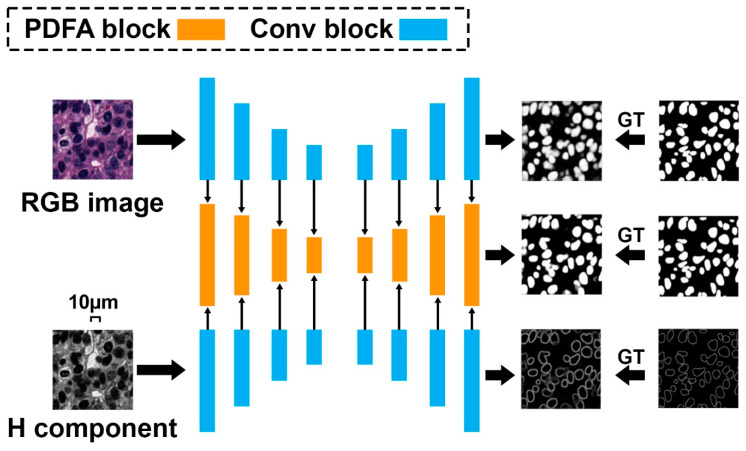
Triple U-Net: hematoxylin-aware nuclei segmentation with progressive dense feature aggregation.

Although the above pixel-wise classification methods have shown more promising performance over the traditional segmentation algorithms, obvious limitations can also be found. First, they are quite slow since the densely selected patches increase the calculation burden for neural network training [80]. Second, the extracted patches cannot fully reveal the rich context information within the whole input image for nuclei segmentation. Accordingly, a more elegant architecture called “fully convolutional network” is proposed [81]. FCN can use the full image rather than the densely extracted patches as the input, which can produce a more accurate and efficient nuclei segmentation result. In addition to FCN, U-Net is another powerful nuclei segmentation tool [82]. In comparison with FCN, U-Net uses skip connections between downsampling and upsampling paths that can stabilize gradient updates for deep model training. Based on the U-Net structure, Zhao et al. [61] proposed a Triple U-Net architecture for nuclei segmentation without the necessity of color normalization and achieved state-of-the-art nuclei segmentation performance (Figure 6). To split touching nuclei that are hard to segment, Yang et al. [60] used a hybrid network consisting of U-Net and region proposal networks, followed by a watershed step to separate them into individual ones. Amirreza et al. [59] proposed a two-stage U-Net–based model for touching cell segmentation, where the first stage used the U-Net to separate nuclei from the background while the second stage applied the U-Net to regress the distance map of each nucleus for the final touching cell segmentation. To explicitly mimic how human pathologists combine multi-scale information, Schmitz et al. [77] introduced a family of multi-encoder FCN with deep fusion for nuclei segmentation. Other U-Net–based studies include [51,58] proposed deep contour-aware networks that integrate multilevel contextual features to accurately detect and segment nuclei from histopathological images, which could also effectively improve the final segmentation performance.

### 4.2. Tissue-Level Segmentation

Besides nuclei segmentation, computerized segmentation of specific tissues in histopathological images is another core operation to study the tumor biology system. For instance, the segmentation of tumor-infiltrating lymphocytes and characterizing their spatial correlation on WSI have become crucial in diagnosis, prognosis, and treatment response prediction for different cancers [83]. Moreover, gland segmentation is one prerequisite step for quantitatively measuring glandular formation, which is also an important indicator for exploring the degree of differentiation [84,85].

The automatic segmentation of tissues in histology images has been explored by many studies [86,87]. Traditional tissue segmentation methods usually relied on the extraction of handcrafted features, the design of conventional classifiers [88]. Recently, deep learning has become popular in computer vision and image-processing tasks due to its outstanding performance, and some studies also applied deep learning methods for the segmentation of different types of tissues from WSI [56,89,90]. Among the existing deep learning segmentation algorithms, the U-Net-based neural network is still most widely used. For example, Saltz et al. [57] applied the U-Net network to present mappings of tumor-infiltrating lymphocytes on H&E images from 13 TCGA (The Cancer Genome Atlas) tumor types. Based on U-Net, Raza et al. [56] presented a minimal information loss dilated network for gland instance segmentation in colon histology images. Chen et al. [89] presented a deep contour-aware network by formulating an explicit contour loss function in the training process and achieved the best performance during the 2015 MICCAI Gland Segmentation (Glas) on-site challenge. Lu et al. [55] proposed BrcaSeg, a WSI processing pipeline that utilized deep learning to perform automatic segmentation and quantification of epithelial and stromal tissues for breast cancer WSI from TCGA. Besides the U-Net structure, Zhao [91] proposed a deep neural network, SCAU-Net, with spatial and channel attention for gland segmentation. SCAU-Net could effectively capture the nonlinear relationship between spatial-wise and channel-wise features, and achieve state-of-the-art gland segmentation performance. Moreover, with the help of the DeeplabV3 model, Musulin [90] developed an enhanced histopathology analysis tool that could accurately segment epithelial and stromal tissue for oral squamous cell carcinoma. Considering that the boundary of the gland is difficult to discriminate, Yan et al. [92] proposed a shape-aware adversarial deep learning framework, which had better tolerance to boundary uncertainty and was more effective for boundary detection. In addition, due to the fixed encoder-decoder structure, U-Net is not suitable for processing texture WSIs, Wen et al. [93] utilized a Gabor-based module to extract texture information at different scales and directions for tissue segmentation. Rojthoven et al. [94] proposed HookNet, a semantic segmentation model combining context information in WSIs via multiple branches of encoder-decoder CNN, for tissue segmentation.

Although much progress has been achieved, the superior performance of previous deep neural network-based methods mainly depends on the substantial number of training images with pixel-wise annotation, which are difficult to obtain due to the requirements of tremendous labeling efforts for experts. In order to reduce the overall labelling cost, several weakly supervised tissue segmentation algorithms have also been proposed [53,95,96]. For instance, Mahapatra [95] proposed a deep active learning framework that could actively select valuable samples from the unlabeled data for annotation, which significantly reduced the annotation efforts while still achieving comparable gland segmentation performance. Lai et al. [96] proposed a semi-supervised active learning framework with a region-based selection criterion. This framework iteratively selects regions for annotation queries to quickly expand the diversity and volume of the labeled set. Besides, Xie et al. [54] proposed a pairwise relation-based semi-supervised model for gland segmentation on histology images, which could produce considerable improvement in learning accuracy with limited labeled images and amounts of unlabeled images. Other studies include [53] having proposed a multiscale conditional GAN for epithelial region segmentation that could be used to compensate for the lack of labeled data in the training dataset. Moreover, Gupta et al. [97] introduced the idea of ‘image enrichment’ whereby the information content of images based on GAN is increased in order to enhance segmentation accuracy.

## 5. Cancer Diagnosis and Prognosis

Cancer is an aggressive disease with a low median survival rate. Ironically, the treatment process is long and very costly due to its high recurrence and mortality rates. Accurate early diagnosis and prognosis prediction of cancer is essential to enhance the patient’s survival rate [98,99]. It is now widely recognized that histopathological images are regarded as golden standards for the diagnosis and prognosis of human cancers [100,101]. Previous studies on histopathology image classification and prediction mainly focused on manual feature design. For instance, Cheng et al. [16] extracted a 150-dimensional handcrafted feature to describe each WSI, followed by the traditional classifiers to distinguish different types of renal cell carcinoma. Yu et al. [102] extracted 9879 quantitative features from each image tile and used regularized machine-learning methods to select the top features and to distinguish shorter-term survivors from longer-term survivors with adenocarcinoma or squamous cell carcinoma. Recently, with the success of deep learning in various computer vision tasks, training end-to-end deep learning models for various histopathological image analysis tasks without manually extracting features has drawn much attention [103,104,105].

**Figure 7:**
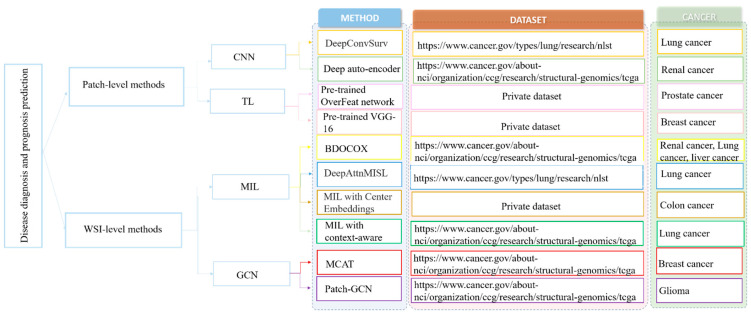
Overview of papers using deep learning for diagnosis and prognosis of the disease in histopathology images [108,109,110,111,112,113,114,115,116].

Generally, the main challenge for applying deep learning algorithms for WSI classification and prediction is the large size of the WSI (e.g., 100,000 × 100,000 pixels), and it is impossible to directly feed these large images into the deep neural network for model training [106,107]. To address this challenge, there are two main lines of approaches, the patch-based and WSI-based methods (which are summarized in Figure 7).

### 5.1. Patch-Level Methods

In connection with the large size of WSI, the patch-based methods required the pathologist to select the region of interests from WSI that are representative, then the selected regions were split into patches with a significantly smaller size for deep model training [108,109,117]. For instance, Zhu et al. [108] developed a deep CNN for survival analysis (DeepConvSurv) with the pathological patches derived from the WSI. They demonstrated that the end-to-end learning algorithm, DeepConvSurv, outperformed the standard Cox proportional hazard model. Cheng et al. [109] applied a deep autoencoder to aggregate the extracted patches into different groups and then learn topological features from the clusters to characterize cell distributions of different cell types for survival prediction.

By considering that training a model from scratch requires a very large dataset and takes a long time to train. Some patch-based methods also adopted the transfer learning model (TL) to speed up the training procedure, as well as improve the classification performance. TL provides an effective solution for feasibly and fast customized accurate models by transferring and fine-tuning the learned knowledge of pre-trained models over large datasets. For instance, Xu et al. [117] exploited CNN activation features to achieve region-level classification results. Specifically, they first over-segmented each preselected region into a set of overlapping patches. A TL strategy was then explored by pretraining CNN with ImageNet. Finally, an SVM classifier was adopted for classification. Similarly, Källénet et al. [110] extracted features from the divided patches via the pre-trained OverFeat network. The RF classifier was applied to discriminate the subtypes in prostatic adenocarcinoma. Moreover, in [111], the pre-trained VGG-16 network was first applied to extract descriptors from the preselected patches. Then, the feature representation of WSI was computed by the average pooling of the feature representations of its associated patches.

### 5.2. WSI-Level Methods

Although much progress has been achieved, the abovementioned patch-level prediction methods still have several inherent drawbacks. First, the patch-based methods required labor-sensitive patch-level annotation, which would increase the workload for the pathologist [118]. Second, most of the existing patch-based methods usually assumed that the diagnosis or survival information with each randomly selected patch was the same as its corresponding WSI, which neglected the fact that WSI usually had large heterogenous patterns and thus the patch-level label would not always match the WSI-level label [119].

**Figure 8:**
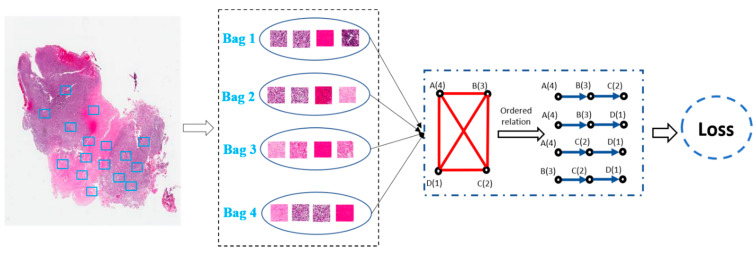
Weakly-supervised deep ordinal Cox model (BDOCOX) for survival prediction from WSI.

In view of these challenges, building diagnosis/prognosis models only relying on WSI-level annotation has been widely investigated [112,119,120]. Among the WSI-based methods, the multi-instance learning (MIL) framework was a simple but most effective tool. For example, Shao et al. [112] considered the ordinal characteristic of the survival process by adding a ranking-based regularization term on the Cox model and used the average pooling strategies to aggregate the instance-level results to the WSI-level prediction results (Figure 8). Similarly, Iizuka et al. [120] first trained a CNN model using millions of tiles extracted from the WSI. Then, a max-pooling strategy combined with the recurrent neural network was adopted to fuse the patch-level results into WSI-level prediction results. However, by considering the simple decision fusion approaches (e.g., average pooling and max pooling) were insufficiently robust to make the right WSI-level prediction, Yao et al. [113] proposed an attention-guided deep multiple instance learning network (DeepAttnMISL) for survival prediction from WSI. In comparison with the traditional pooling strategies, attention-based aggregation is more flexible and adaptive for survival prediction. In addition, Chikontwe et al. [114] presented a novel MIL framework for histopathology slide classification. The proposed framework could be applied for both instance and bag level learning with a center loss that minimized intraclass distances in the embedding space. The experimental results also suggested that the proposed method could achieve overall improved performance over recent state-of-the-art methods. Moreover, Wang et al. [119] first extracted the spatial contextual features from each patch. Then, a globally holistic region descriptor was calculated after aggregating the features from multiple representative instances for WSI-level classification.

Although CNN-based MIL frameworks have shown impressive performance in the field of histopathology analysis, they are unable to capture complex neighborhood information as they analyze local areas determined by the convolutional kernel to extract interaction information between objects. Recently, some researchers have also applied the graph convolutional network (GCN) to analyze histopathological images for the diagnosis and prognosis of human cancers [115,121], which are becoming increasingly useful for medical diagnosis and prognosis. For instance, Chen et al. [115] presented a context-aware graph convolutional network that hierarchically aggregates instance-level histology features to model local- and global-level topological structures in the tumor microenvironment. Li et al. [121] proposed to model WSI as a graph and then develop a graph convolutional neural network with attention learning that better serves the survival prediction by rendering the optimal graph representations of WSIs. Moreover, the study in [122] presented a patch relevance-enhanced graph convolutional network (RGCN) to explicitly model the correlations of different patches in WSI, which can approximately estimate the diagnosis-related regions in WSI. Extensive experiments on real lung and brain carcinoma WSIs have demonstrated their effectiveness since GCNs can better exploit and preserve neighboring relations compared with CNN-based models. Besides, some researchers have noticed the relation between genes and images. Chen et al. [116] presented a multimodal co-attention transformer (MCAT) framework that learns an interpretable, dense co-attention mapping between WSI and genomic features formulated in an embedding space.

## 6. Open Resources and Future Work

### 6.1. Open Resources

A collection of high-quality labeled datasets is an important prerequisite for deep model training. We show the benchmark datasets in terms of different tasks in Table 2. Specifically, to carry out color normalization tasks, NIA Lymphoma 2009, UCSB, CAMELYON16, and CAMELYON17 datasets were most widely used. As for nuclei/tissue segmentation tasks, MoNuSeg 2018, TNBC 2018, GLAS 2015, and CRAG 2019 projects provided essential information for the convenience of deep model training. Finally, the datasets of ACDC-LungHP 2019, CRCHisto 2016, and CoNSeP 2019 collected the WSI and their corresponding diagnosis/prognosis information for numerous cancers patients. As can be seen from Table 3, QuPath [123], PMA.start, Orbit [124], and CellProfiler [125] are open, powerful, flexible, extensible software platforms for bioimage analysis, which can conduct each step for pathological image analysis. Openslide [126] is a Python package that can provide a simple interface to read WSI, and ASAP is an open-source WSI viewer which focuses on fast and fluid image viewing with an easy-to-use interface for making annotations based on Openslide. In addition, ImageJ [127] is also a famous open-source medical imaging viewer which can add powerful plug-ins to use many image analysis algorithms. A plugin for ImageJ, named SlideJ, can seamlessly extend the application of image analysis algorithms implemented in ImageJ for single microscopic field images to a WSI analysis. Finally, The Cytomine software [128] is an open-source web platform that can foster collaborative analysis of very large images and allows for semi-automatic processing of large image collections via machine learning algorithms.

### 6.2. Future Work

We primarily reviewed the recently developed deep learning algorithms employed for the analysis of histopathological images. Although tremendous efforts have been made, several issues should be addressed in future studies. First, most color normalization algorithms are designed to match the H&E-stained images derived from different sources. However, it is still challenging to accomplish the color transformation task from H&E-stained images to other immunohistochemistry-stained images due to the large variance between them. Applying the normalization step to match the image with different stains that can facilitate a chromatic distinction among different tissue constituents needs more study [129]. Second, although the deep learning algorithms have shown their advantages for the segmentation of nuclei and specific tissues from the histopathological image, the generation of an adequate volume of high-quality labels still needs tremendous annotation efforts from the pathologist. While the existing weakly supervised learning algorithms, such as active learning and semi-supervised learning methods, can reduce the annotation workload on pathologists to some extent, a design for a scalable crowdsourcing approach [130] that benefits from the participation of non-pathologists to reduce pathologist effort and enables minimal-effort collection of segmentation boundaries is needed. Third, most of the WSI-level diagnosis or prognosis models are calculated in a black box, so that no human can understand which part in the WSI mostly affects the final prediction. To make our model more explainable, it is desirable to design a deep learning model that can identify discriminant patches from the WSI that triggers the clinical results. Finally, imaging genomics [131], as an emerging research field, has also created new opportunities for the diagnosis and prognosis of human cancers. How to effectively combine the imaging and genomic data [132] to help better understand prognostic and, hopefully, therapeutic aspects of various human cancers is another interesting and prospective research direction in the future.

## 7. Conclusions

We have reviewed the advanced deep learning algorithms for the computational analysis of H&E-stained histopathological images. We presented some recent findings on the state-of-the-art deep learning techniques on different H&E-stained pathological image analysis tasks, such as color normalization, nuclei/tissue segmentation, and the diagnosis and prognosis of human cancers. We also provided online resources and outlined open research problems on digital H&E-stained pathology image analysis. Deep learning is a powerful tool, providing reliable support for diagnostic assessment and treatment decisions. Last but not least, we also provided open research problems for future studies including removing the stain variation between H/E and IHC stained images, reducing the human annotation efforts for tissue/nuclei segmentation, designing the explainable deep neural network for identifying discriminant and meaningful patches from the image, and integrating histopathological images with genomic data for clinical outcome prediction.

## Figures and Tables

**Table 1:** Comparison of convolution neural networks for computer vision.

Model	AlexNet [28]	ZFNet [29]	VGGNet-19 [30]	GoogLeNet [31]	ResNet-152 [32]	SENET [33]
Input size	227 × 227	224 × 224	224 × 224	224 × 224	224 × 224	224 × 224
Top-5 error(%)	15.3	11.2	7.50	6.67	3.57	97.75
Layer number	8	8	19	22	152	152
Convolution layer number	5	5	16	21	151	151
Kernel size	11, 5, 3	7, 5, 3	3	7, 1, 3, 5	7, 1, 3, 5	7, 1, 3, 5
Full connected layer number	3	3	3	1	1	1
Model size	60 M	140 M	144 M	500 M	60 M	64 M
Calculation speed	727 M	1.6 G	20 G	2 G	11 G	21 G
Dropout	√	√	√	√	√	√
Batch Normalization	×	×	×	×	√	√

**Table 2:** Summary of publicly available databases in computational histopathology.

ID	Cancer Types	Images/Cases	Link
Color normalization			
NIA Lymphoma 2009	lymphoma	375	https://www.nia.nih.gov (accessed on 17 January 2022)
UCSB	Breast	58	http://iridl.ldeo.columbia.edu/SOURCES/.UCSB/ (accessed on 17 January 2022)
CAMELYON16 2016	Breast	400	https://camelyon16.grand-challenge.org/ (accessed on 17 January 2022)
CAMELYON17 2017	Breast	1000	https://camelyon17.grand-challenge.org/ (accessed on 17 January 2022)
Pathology image segmentation			
Nuclei segmentation			
MoNuSeg 2018	Multi-tissue	44	https://monuseg.grand-challenge.org/Home/ (accessed on 17 January 2022)
TNBC 2018	Breast	50	https://github.com/PeterJackNaylor/DRFNS (accessed on 17 January 2022)
Gland segmentation			
GLAS 2015	Colon	165	https://warwick.ac.uk/fac/sci/dcs/research/tia/glascontest/ (accessed on 17 January 2022)
CRAG 2019	Colon	213	https://warwick.ac.uk/fac/cross_fac/tia/data/mildnet/ (accessed on 17 January 2022)
Diagnosis and prognosis			
Diagnosis			
ICPR 2014	Breast	2112	https://mitos-atypia-14.grand-challenge.org/ (accessed on 17 January 2022)
BreakHis 2016	Breast	82	https://mitos-atypia-14.grand-challenge.org/ (accessed on 17 January 2022)
HER2 Scoring 2016	Breast	86	https://warwick.ac.uk/fac/sci/dcs/research/tia/her2contest/ (accessed on 17 January 2022)
BACH 2018	Breast	500	https://web.inf.ufpr.br/vri/databases/breast-cancer-histopathological-database-breakhis/ (accessed on 17 January 2022)
Prognosis			
CRCHisto 2016	Colon	100	https://warwick.ac.uk/fac/cross_fac/tia/data/crchistolabelednucleihe/ (accessed on 17 January 2022)
NCT-CRC-HE-100k 2019	Colon	100,000	https://zenodo.org/record/1214456#.YeV8MnpByUl (accessed on 17 January 2022)
ACDC-LungHP 2019	Lung	200	https://acdc-lunghp.grand-challenge.org/ (accessed on 17 January 2022)
CoNSeP 2019	Colon	41	https://warwick.ac.uk/fac/cross_fac/tia/data/hovernet/ (accessed on 17 January 2022)
Multiple			
TCIA	Multi-cancer	-	https://www.cancerimagingarchive.net/ (accessed on 17 January 2022)

**Table 3:** Summary of publicly available tools in computational histopathology.

Tool Name	Language	View	Color Normalization	Segmentation	Diagnosis /Prognosis	Link	Reference
Qupath	Java	√	√	√	√	https://qupath.github.io/ (accessed on 17 January 2022)	[123]
Cytomine	Java, web	√	×	√	√	https://cytomine.be/ (accessed on 17 January 2022)	[128]
Orbit	Java, Scala, Python, R, and SQL	√	√	√	√	https://www.orbit.bio/ (accessed on 17 January 2022)	[124]
ASAP	Python	√	×	×	×	https://computationalpathologygroup.github.io/ASAP/ (accessed on 17 January 2022)	\
Openslide	C, Java	√	√	√	√	https://openslide.org/demo/ (accessed on 17 January 2022)	[126]
ImageJ	Java	√	√	√	√	https://imagej.net/plugins/slidej (accessed on 17 January 2022)	[127]
PMA.start	Web	√	√	√	√	https://free.pathomation.com/ (accessed on 17 January 2022)	\
CellProfiler	Python	√	√	√	√	https://cellprofiler.org/ (accessed on 17 January 2022)	[125]

## References

[1] Sung H., Ferlay J., Siegel R.L., Laversanne M., Soerjomataram I., Jemal A., Bray F. (2021). Global cancer statistics 2020: GLOBOCAN estimates of incidence and mortality worldwide for 36 cancers in 185 countries. CA A Cancer J. Clin..

[2] Cai X., Li X., Razmjooy N., Ghadimi N. (2021). Breast Cancer Diagnosis by Convolutional Neural Network and Advanced Thermal Exchange Optimization Algorithm. Comput. Math. Methods Med..

[3] Shao W., Han Z., Cheng J., Cheng L., Wang T., Sun L., Lu Z., Zhang J., Zhang D., Huang K. (2020). Integrative Analysis of Pathological Images and Multi-Dimensional Genomic Data for Early-Stage Cancer Prognosis. IEEE Trans. Med. Imaging.

[4] Xu J., Xiang L., Liu Q., Gilmore H., Wu J., Tang J., Madabhushi A. (2016). Stacked Sparse Autoencoder (SSAE) for Nuclei Detection on Breast Cancer Histopathology Images. IEEE Trans. Med. Imaging.

[5] Stacke K., Eilertsen G., Unger J., Lundström C. (2020). Measuring domain shift for deep learning in histopathology. IEEE J. Biomed. Health Inform..

[6] Zhu W., Xie L., Han J., Guo X. (2020). The application of deep learning in cancer prognosis prediction. Cancers.

[7] Peikari M., Gangeh M.J., Zubovits J., Clarke G., Martel A.L. (2015). Triaging diagnostically relevant regions from pathology whole slides of breast cancer: A texture based approach. IEEE Trans. Med. Imaging.

[8] Anoraganingrum D. Cell segmentation with median filter and mathematical morphology operation. Proceedings of the 10th International Conference on Image Analysis and Processing.

[9] Platt J. (1998). Sequential Minimal Optimization: A Fast Algorithm for Training Support Vector Machines. https://www.microsoft.com/en-us/research/publication/sequential-minimal-optimization-a-fast-algorithm-for-training-support-vector-machines/.

[10] Breiman L. (2001). Random forests. Mach. Learn..

[11] Dudani S.A. (1976). The distance-weighted k-nearest-neighbor rule. IEEE Trans. Syst. Man Cybern..

[12] Kruk M., Kurek J., Osowski S., Koktysz R., Swiderski B., Markiewicz T. (2017). Ensemble of classifiers and wavelet transformation for improved recognition of Fuhrman grading in clear-cell renal carcinoma. Biocybern. Biomed. Eng..

[13] Fuchs T.J., Wild P.J., Moch H., Buhmann J.M. Computational pathology analysis of tissue microarrays predicts survival of renal clear cell carcinoma patients. Proceedings of the International Conference on Medical Image Computing and Computer-Assisted Intervention.

[14] Zarella M.D., Yeoh C., Breen D.E., Garcia F.U. (2017). An alternative reference space for H&E color normalization. PLoS ONE.

[15] Freitag D. Information extraction from HTML: Application of a general machine learning approach. Proceedings of the AAAI/IAAI.

[16] Cheng J., Han Z., Mehra R., Shao W., Cheng M., Feng Q., Ni D., Huang K., Cheng L., Zhang J. (2020). Computational analysis of pathological images enables a better diagnosis of TFE3 Xp11. 2 translocation renal cell carcinoma. Nat. Commun..

[17] Mao B., Zhang L., Ning P., Ding F., Wu F., Lu G., Geng Y., Ma J. (2020). Preoperative prediction for pathological grade of hepatocellular carcinoma via machine learning–based radiomics. Eur. Radiol..

[18] Cosatto E., Laquerre P.-F., Malon C., Graf H.-P., Saito A., Kiyuna T., Marugame A., Kamijo K.I. Automated gastric cancer diagnosis on h&e-stained sections; ltraining a classifier on a large scale with multiple instance machine learning. Proceedings of the Medical Imaging 2013: Digital Pathology.

[19] Komura D., Ishikawa S. (2019). Machine learning approaches for pathologic diagnosis. Virchows Arch..

[20] Jimenez-del-Toro O., Otálora S., Andersson M., Eurén K., Hedlund M., Rousson M., Müller H., Atzori M. (2017). Analysis of histopathology images: From traditional machine learning to deep learning. Biomedical Texture Analysis.

[21] Pasquini G., Arias J.E.R., Schäfer P., Busskamp V. (2021). Automated methods for cell type annotation on scRNA-seq data. Comput. Struct. Biotechnol. J..

[22] van der Laak J., Litjens G., Ciompi F. (2021). Deep learning in histopathology: The path to the clinic. Nat. Med..

[23] Goldenberg S.L., Nir G., Salcudean S.E. (2019). A new era: Artificial intelligence and machine learning in prostate cancer. Nat. Rev. Urol..

[24] Fakoor R., Ladhak F., Nazi A., Huber M. Using deep learning to enhance cancer diagnosis and classification. Proceedings of the International Conference on Machine Learning.

[25] Zhao Z.-Q., Zheng P., Xu S.-T., Wu X. (2019). Object detection with deep learning: A review. IEEE Trans. Neural Netw. Learn. Syst..

[26] Garcia-Garcia A., Orts-Escolano S., Oprea S., Villena-Martinez V., Garcia-Rodriguez J. (2017). A review on deep learning techniques applied to semantic segmentation. arXiv.

[27] Li Y., Hao Z., Lei H. (2016). Survey of convolutional neural network. J. Comput. Appl..

[28] Krizhevsky A., Sutskever I., Hinton G.E. (2012). Imagenet classification with deep convolutional neural networks. Adv. Neural Inf. Process. Syst..

[29] Zeiler M.D., Fergus R. Visualizing and understanding convolutional networks. Proceedings of the European Conference on Computer Vision.

[30] Simonyan K., Zisserman A. (2014). Very deep convolutional networks for large-scale image recognition. arXiv.

[31] Szegedy C., Liu W., Jia Y., Sermanet P., Reed S., Anguelov D., Erhan D., Vanhoucke V., Rabinovich A. Going deeper with convolutions. Proceedings of the IEEE Conference on Computer Vision and Pattern Recognition.

[32] He K., Zhang X., Ren S., Sun J. Deep residual learning for image recognition. Proceedings of the IEEE Conference on Computer Vision and Pattern Recognition.

[33] Hu J., Shen L., Sun G. Squeeze-and-excitation networks. Proceedings of the IEEE Conference on Computer Vision and Pattern Recognition.

[34] Krithiga R., Geetha P. (2021). Breast cancer detection, segmentation and classification on histopathology images analysis: A systematic review. Arch. Comput. Methods Eng..

[35] Shaban M.T., Baur C., Navab N., Albarqouni S. Staingan: Stain style transfer for digital histological images. Proceedings of the 2019 IEEE 16th international symposium on biomedical imaging (ISBI 2019).

[36] de Bel T., Hermsen M., Kers J., van der Laak J., Litjens G. Stain-transforming cycle-consistent generative adversarial networks for improved segmentation of renal histopathology. Proceedings of the International Conference on Medical Imaging with Deep Learning–Full Paper Track.

[37] Bentaieb A., Hamarneh G. (2018). Adversarial Stain Transfer for Histopathology Image Analysis. IEEE Trans. Med. Imaging.

[38] Mahapatra D., Bozorgtabar B., Thiran J.-P., Shao L. Structure preserving stain normalization of histopathology images using self supervised semantic guidance. Proceedings of the International Conference on Medical Image Computing and Computer-Assisted Intervention.

[39] Cong C., Liu S., Di Ieva A., Pagnucco M., Berkovsky S., Song Y. Texture Enhanced Generative Adversarial Network For Stain Normalisation In Histopathology Images. Proceedings of the 2021 IEEE 18th International Symposium on Biomedical Imaging (ISBI).

[40] Patil A., Talha M., Bhatia A., Kurian N.C., Mangale S., Patel S., Sethi A. Fast, Self Supervised, Fully Convolutional Color Normalization Of H&E Stained Images. Proceedings of the 2021 IEEE 18th International Symposium on Biomedical Imaging (ISBI).

[41] Janowczyk A., Basavanhally A., Madabhushi A. (2017). Stain Normalization using Sparse AutoEncoders (StaNoSA): Application to digital pathology. Comput. Med. Imaging Graph..

[42] Ruifrok A.C., Johnston D.A. (2001). Quantification of histochemical staining by color deconvolution. Anal. Quant. Cytol. Histol..

[43] Reinhard E., Ashikhmin N., Gooch B., Shirley P. (2001). Color transfer between images. IEEE Comput. Graph. Appl..

[44] Vahadane A., Peng T., Sethi A., Albarqouni S., Wang L., Baust M., Steiger K., Schlitter A.M., Esposito I., Navab N. (2016). Structure-Preserving Color Normalization and Sparse Stain Separation for Histological Images. IEEE. Trans. Med. Imaging.

[45] Bug D., Schneider S., Grote A., Oswald E., Feuerhake F., Schüler J., Merhof D. (2017). Context-based normalization of histological stains using deep convolutional features. Deep Learning in Medical Image Analysis and Multimodal Learning for Clinical Decision Support.

[46] Zanjani F.G., Zinger S., Bejnordi B.E., van der Laak J.A., de With P.H. Stain normalization of histopathology images using generative adversarial networks. Proceedings of the 2018 IEEE 15th International Symposium on Biomedical Imaging (ISBI 2018).

[47] Zhu J.-Y., Park T., Isola P., Efros A.A. Unpaired image-to-image translation using cycle-consistent adversarial networks. Proceedings of the IEEE International Conference on Computer Vision.

[48] Chan L., Hosseini M.S., Rowsell C., Plataniotis K.N., Damaskinos S. Histosegnet: Semantic segmentation of histological tissue type in whole slide images. Proceedings of the IEEE/CVF International Conference on Computer Vision.

[49] Zhang H., Liu J., Yu Z., Wang P. (2021). MASG-GAN: A multi-view attention superpixel-guided generative adversarial network for efficient and simultaneous histopathology image segmentation and classification. Neurocomputing.

[50] Sucher R., Sucher E. (2020). Artificial intelligence is poised to revolutionize human liver allocation and decrease medical costs associated with liver transplantation. HepatoBiliary Surg. Nutr..

[51] Chen H., Qi X., Yu L., Dou Q., Qin J., Heng P.A. (2017). DCAN: Deep contour-aware networks for object instance segmentation from histology images. Med. Image Anal..

[52] Janowczyk A., Madabhushi A. (2016). Deep learning for digital pathology image analysis: A comprehensive tutorial with selected use cases. J. Pathol. Inform..

[53] Li W., Li J., Polson J., Wang Z., Speier W., Arnold C. (2022). High resolution histopathology image generation and segmentation through adversarial training. Med. Image Anal.

[54] Xie Y., Zhang J., Liao Z., Verjans J., Shen C., Xia Y. Pairwise relation learning for semi-supervised gland segmentation. Proceedings of the International Conference on Medical Image Computing and Computer-Assisted Intervention.

[55] Lu Z., Zhan X., Wu Y., Cheng J., Shao W., Ni D., Han Z., Zhang J., Feng Q., Huang K. (2021). BrcaSeg: A Deep Learning Approach for Tissue Quantification and Genomic Correlations of Histopathological Images. Genom. Proteom. Bioinform..

[56] Raza S.E.A., Cheung L., Epstein D., Pelengaris S., Khan M., Rajpoot N.M. Mimo-net: A multi-input multi-output convolutional neural network for cell segmentation in fluorescence microscopy images. Proceedings of the 2017 IEEE 14th international symposium on biomedical imaging (ISBI 2017).

[57] Saltz J., Gupta R., Hou L., Kurc T., Singh P., Nguyen V., Samaras D., Shroyer K.R., Zhao T., Batiste R. (2018). Spatial Organization and Molecular Correlation of Tumor-Infiltrating Lymphocytes Using Deep Learning on Pathology Images. Cell Rep..

[58] Samanta P., Raipuria G., Singhal N. Context Aggregation Network For Semantic Labeling In Histopathology Images. Proceedings of the 2021 IEEE 18th International Symposium on Biomedical Imaging (ISBI).

[59] Mahbod A., Schaefer G., Ellinger I., Ecker R., Smedby Ö., Wang C. A two-stage U-Net algorithm for segmentation of nuclei in H&E-stained tissues. Proceedings of the European Congress on Digital Pathology.

[60] Yang L., Ghosh R.P., Franklin J.M., Chen S., You C., Narayan R.R., Melcher M.L., Liphardt J.T. (2020). NuSeT: A deep learning tool for reliably separating and analyzing crowded cells. PLoS Comput. Biol..

[61] Zhao B., Chen X., Li Z., Yu Z., Yao S., Yan L., Wang Y., Liu Z., Liang C., Han C. (2020). Triple U-net: Hematoxylin-aware nuclei segmentation with progressive dense feature aggregation. Med. Image Anal..

[62] Song Y., Zhang L., Chen S., Ni D., Lei B., Wang T. (2015). Accurate Segmentation of Cervical Cytoplasm and Nuclei Based on Multiscale Convolutional Network and Graph Partitioning. IEEE Trans. Biomed. Eng..

[63] Zhou Y., Chang H., Barner K.E., Parvin B. Nuclei segmentation via sparsity constrained convolutional regression. Proceedings of the 2015 IEEE 12th International Symposium on Biomedical Imaging (ISBI).

[64] Ciresan D., Giusti A., Gambardella L., Schmidhuber J. (2012). Deep neural networks segment neuronal membranes in electron microscopy images. Adv. Neural Inf Process. Syst..

[65] Qi X., Xing F., Foran D.J., Yang L. (2012). Robust segmentation of overlapping cells in histopathology specimens using parallel seed detection and repulsive level set. IEEE Trans. Biomed. Eng..

[66] Veta M., van Diest P.J., Willems S.M., Wang H., Madabhushi A., Cruz-Roa A., Gonzalez F., Larsen A.B., Vestergaard J.S., Dahl A.B. (2015). Assessment of algorithms for mitosis detection in breast cancer histopathology images. Med. Image Anal..

[67] Oren A., Fernandes J. (1991). The Bethesda system for the reporting of cervical/vaginal cytology. J. Am. Osteopath. Assoc..

[68] Liu T., Li G., Nie J., Tarokh A., Zhou X., Guo L., Malicki J., Xia W., Wong S.T. (2008). An automated method for cell detection in zebrafish. Neuroinformatics.

[69] Lu Z., Carneiro G., Bradley A.P. (2015). An improved joint optimization of multiple level set functions for the segmentation of overlapping cervical cells. IEEE Trans. Image Process..

[70] Dorini L.B., Minetto R., Leite N.J. (2012). Semiautomatic white blood cell segmentation based on multiscale analysis. IEEE J. Biomed. Health Inform..

[71] Zhang C., Yarkony J., Hamprecht F.A. Cell detection and segmentation using correlation clustering. Proceedings of the International Conference on Medical Image Computing and Computer-Assisted Intervention.

[72] Bergeest J.-P., Rohr K. (2012). Efficient globally optimal segmentation of cells in fluorescence microscopy images using level sets and convex energy functionals. Med. Image Anal..

[73] Sahara K., Paredes A.Z., Tsilimigras D.I., Sasaki K., Moro A., Hyer J.M., Mehta R., Farooq S.A., Wu L., Endo I. (2021). Machine learning predicts unpredicted deaths with high accuracy following hepatopancreatic surgery. Hepatobiliary Surg. Nutr..

[74] Mahmood F., Borders D., Chen R.J., McKay G.N., Salimian K.J., Baras A., Durr N.J. (2019). Deep adversarial training for multi-organ nuclei segmentation in histopathology images. IEEE Trans. Med. Imaging.

[75] Liu D., Zhang D., Song Y., Zhang C., Zhang F., O’Donnell L., Cai W. Nuclei Segmentation via a Deep Panoptic Model with Semantic Feature Fusion. Proceedings of the 2019 International Joint Conference on Artificial Intelligence.

[76] Moris D., Shaw B.I., Ong C., Connor A., Samoylova M.L., Kesseli S.J., Abraham N., Gloria J., Schmitz R., Fitch Z.W. (2021). A simple scoring system to estimate perioperative mortality following liver resection for primary liver malignancy—the Hepatectomy Risk Score (HeRS). Hepatobiliary Surg. Nutr..

[77] Schmitz R., Madesta F., Nielsen M., Krause J., Steurer S., Werner R., Rösch T. (2021). Multi-scale fully convolutional neural networks for histopathology image segmentation: From nuclear aberrations to the global tissue architecture. Med. Image Anal..

[78] Xing F., Xie Y., Yang L. (2015). An automatic learning-based framework for robust nucleus segmentation. IEEE Trans. Med. Imaging.

[79] Settouti N., Bechar M.E., Daho M.E., Chikh M.A. (2020). An optimised pixel-based classification approach for automatic white blood cells segmentation. Int. J. Biomed. Eng. Technol..

[80] Sahasrabudhe M., Christodoulidis S., Salgado R., Michiels S., Loi S., André F., Paragios N., Vakalopoulou M. Self-supervised nuclei segmentation in histopathological images using attention. Proceedings of the International Conference on Medical Image Computing and Computer-Assisted Intervention.

[81] Long J., Shelhamer E., Darrell T. Fully convolutional networks for semantic segmentation. Proceedings of the IEEE Conference on Computer Vision and Pattern Recognition.

[82] Ronneberger O., Fischer P., Brox T. U-net: Convolutional networks for biomedical image segmentation. Proceedings of the International Conference on Medical Image Computing and Computer-Assisted Intervention.

[83] Denkert C., Loibl S., Noske A., Roller M., Muller B.M., Komor M., Budczies J., Darb-Esfahani S., Kronenwett R., Hanusch C. (2010). Tumor-associated lymphocytes as an independent predictor of response to neoadjuvant chemotherapy in breast cancer. J. Clin. Oncol..

[84] Sirinukunwattana K., Pluim J.P.W., Chen H., Qi X., Heng P.A., Guo Y.B., Wang L.Y., Matuszewski B.J., Bruni E., Sanchez U. (2017). Gland segmentation in colon histology images: The glas challenge contest. Med. Image Anal..

[85] Vukicevic A.M., Radovic M., Zabotti A., Milic V., Hocevar A., Callegher S.Z., De Lucia O., De Vita S., Filipovic N. (2021). Deep learning segmentation of Primary Sjögren’s syndrome affected salivary glands from ultrasonography images. Comput. Biol. Med..

[86] Gunduz-Demir C., Kandemir M., Tosun A.B., Sokmensuer C. (2010). Automatic segmentation of colon glands using object-graphs. Med. Image Anal..

[87] Salvi M., Bosco M., Molinaro L., Gambella A., Papotti M., Acharya U.R., Molinari F. (2021). A hybrid deep learning approach for gland segmentation in prostate histopathological images. Artif. Intell. Med..

[88] Fleming M., Ravula S., Tatishchev S.F., Wang H.L. (2012). Colorectal carcinoma: Pathologic aspects. J. Gastrointest. Oncol..

[89] Chen H., Qi X., Yu L., Heng P.-A. DCAN: Deep contour-aware networks for accurate gland segmentation. Proceedings of the IEEE conference on Computer Vision and Pattern Recognition.

[90] Musulin J., Stifanic D., Zulijani A., Cabov T., Dekanic A., Car Z. (2021). An Enhanced Histopathology Analysis: An AI-Based System for Multiclass Grading of Oral Squamous Cell Carcinoma and Segmenting of Epithelial and Stromal Tissue. Cancers.

[91] Zhao P., Zhang J., Fang W., Deng S. (2020). SCAU-Net: Spatial-Channel Attention U-Net for Gland Segmentation. Front. Bioeng. Biotechnol..

[92] Yan Z., Yang X., Cheng K.T. (2020). Enabling a Single Deep Learning Model for Accurate Gland Instance Segmentation: A Shape-Aware Adversarial Learning Framework. IEEE Trans. Med. Imaging.

[93] Wen Z., Feng R., Liu J., Li Y., Ying S. (2020). GCSBA-Net: Gabor-Based and Cascade Squeeze Bi-Attention Network for Gland Segmentation. IEEE J. Biomed. Health Inform..

[94] van Rijthoven M., Balkenhol M., Siliņa K., van der Laak J., Ciompi F. (2021). HookNet: Multi-resolution convolutional neural networks for semantic segmentation in histopathology whole-slide images. Med. Image Anal..

[95] Mahapatra D., Poellinger A., Shao L., Reyes M. (2021). Interpretability-Driven Sample Selection Using Self Supervised Learning for Disease Classification and Segmentation. IEEE Trans. Med. Imaging.

[96] Lai Z., Wang C., Oliveira L.C., Dugger B.N., Cheung S.-C., Chuah C.-N. Joint Semi-supervised and Active Learning for Segmentation of Gigapixel Pathology Images with Cost-Effective Labeling. Proceedings of the Proceedings of the IEEE/CVF International Conference on Computer Vision.

[97] Gupta L., Klinkhammer B.M., Boor P., Merhof D., Gadermayr M. GAN-based image enrichment in digital pathology boosts segmentation accuracy. Proceedings of the International Conference on Medical Image Computing and Computer-Assisted Intervention.

[98] Hu A., Razmjooy N. (2021). Brain tumor diagnosis based on metaheuristics and deep learning. Int. J. Imaging Syst. Technol..

[99] Shen X., Zhao H., Jin X., Chen J., Yu Z., Ramen K., Zheng X., Wu X., Shan Y., Bai J. (2021). Development and validation of a machine learning-based nomogram for prediction of intrahepatic cholangiocarcinoma in patients with intrahepatic lithiasis. Hepatobiliary Surg. Nutr..

[100] Huang S., Yang J., Fong S., Zhao Q. (2020). Artificial intelligence in cancer diagnosis and prognosis: Opportunities and challenges. Cancer Lett..

[101] Lau W.Y., Wang K., Zhang X.P., Li L.Q., Wen T.F., Chen M.S., Jia W.D., Xu L., Shi J., Guo W.X. (2021). A new staging system for hepatocellular carcinoma associated with portal vein tumor thrombus. Hepatobiliary Surg. Nutr..

[102] Yu K.H., Zhang C., Berry G.J., Altman R.B., Re C., Rubin D.L., Snyder M. (2016). Predicting non-small cell lung cancer prognosis by fully automated microscopic pathology image features. Nat. Commun..

[103] Hosseini M.S., Brawley-Hayes J.A., Zhang Y., Chan L., Plataniotis K.N., Damaskinos S. (2019). Focus quality assessment of high-throughput whole slide imaging in digital pathology. IEEE Trans. Med. Imaging.

[104] Yao J., Zhu X., Huang J. Deep multi-instance learning for survival prediction from whole slide images. Proceedings of the International Conference on Medical Image Computing and Computer-Assisted Intervention.

[105] Yang H., Kim J.-Y., Kim H., Adhikari S.P. (2019). Guided soft attention network for classification of breast cancer histopathology images. IEEE Trans. Med. Imaging.

[106] Chen C., Lu M.Y., Williamson D.F., Chen T.Y., Schaumberg A.J., Mahmood F. (2021). Fast and Scalable Image Search For Histology. arXiv.

[107] Sun H., Zeng X., Xu T., Peng G., Ma Y. (2019). Computer-aided diagnosis in histopathological images of the endometrium using a convolutional neural network and attention mechanisms. IEEE J. Biomed. Health Inform..

[108] Zhu X., Yao J., Huang J. Deep convolutional neural network for survival analysis with pathological images. Proceedings of the 2016 IEEE International Conference on Bioinformatics and Biomedicine (BIBM).

[109] Cheng J., Mo X., Wang X., Parwani A., Feng Q., Huang K. (2018). Identification of topological features in renal tumor microenvironment associated with patient survival. Bioinformatics.

[110] Källén H., Molin J., Heyden A., Lundström C., Åström K. Towards grading gleason score using generically trained deep convolutional neural networks. Proceedings of the 2016 IEEE 13th International Symposium on Biomedical Imaging (ISBI).

[111] Mercan C., Aksoy S., Mercan E., Shapiro L.G., Weaver D.L., Elmore J.G. From patch-level to ROI-level deep feature representations for breast histopathology classification. Proceedings of the Medical Imaging 2019: Digital Pathology.

[112] Shao W., Wang T., Huang Z., Han Z., Zhang J., Huang K. (2021). Weakly supervised deep ordinal cox model for survival prediction from whole-slide pathological images. IEEE Trans. Med. Imaging.

[113] Yao J., Zhu X., Jonnagaddala J., Hawkins N., Huang J. (2020). Whole slide images based cancer survival prediction using attention guided deep multiple instance learning networks. Med. Image Anal..

[114] Chikontwe P., Kim M., Nam S.J., Go H., Park S.H. Multiple instance learning with center embeddings for histopathology classification. Proceedings of the International Conference on Medical Image Computing and Computer-Assisted Intervention.

[115] Chen R.J., Lu M.Y., Shaban M., Chen C., Chen T.Y., Williamson D.F., Mahmood F. Whole Slide Images are 2D Point Clouds: Context-Aware Survival Prediction using Patch-based Graph Convolutional Networks. Proceedings of the International Conference on Medical Image Computing and Computer-Assisted Intervention.

[116] Chen R.J., Lu M.Y., Weng W.-H., Chen T.Y., Williamson D.F., Manz T., Shady M., Mahmood F. Multimodal Co-Attention Transformer for Survival Prediction in Gigapixel Whole Slide Images. Proceedings of the IEEE/CVF International Conference on Computer Vision.

[117] Xu Y., Jia Z., Wang L.B., Ai Y., Zhang F., Lai M., Chang E.I. (2017). Large scale tissue histopathology image classification, segmentation, and visualization via deep convolutional activation features. BMC Bioinform..

[118] Marini N., Otalora S., Muller H., Atzori M. (2021). Semi-supervised training of deep convolutional neural networks with heterogeneous data and few local annotations: An experiment on prostate histopathology image classification. Med. Image Anal..

[119] Wang X., Chen H., Gan C., Lin H., Dou Q., Tsougenis E., Huang Q., Cai M., Heng P.A. (2020). Weakly Supervised Deep Learning for Whole Slide Lung Cancer Image Analysis. IEEE Trans. Cybern..

[120] Iizuka O., Kanavati F., Kato K., Rambeau M., Arihiro K., Tsuneki M. (2020). Deep Learning Models for Histopathological Classification of Gastric and Colonic Epithelial Tumours. Sci. Rep..

[121] Li R., Yao J., Zhu X., Li Y., Huang J. Graph CNN for survival analysis on whole slide pathological images. Proceedings of the International Conference on Medical Image Computing and Computer-Assisted Intervention.

[122] Chen Z., Zhang J., Che S., Huang J., Han X., Yuan Y. Diagnose Like A Pathologist: Weakly-Supervised Pathologist-Tree Network for Slide-Level Immunohistochemical Scoring. Proceedings of the 35th AAAI Conference on Artificial Intelligence (AAAI-21).

[123] Bankhead P., Loughrey M.B., Fernández J.A., Dombrowski Y., McArt D.G., Dunne P.D., McQuaid S., Gray R.T., Murray L.J., Coleman H.G. (2017). QuPath: Open source software for digital pathology image analysis. Sci. Rep..

[124] Stritt M., Stalder A.K., Vezzali E. (2020). Orbit image analysis: An open-source whole slide image analysis tool. PLoS Comput. Biol..

[125] Carpenter A.E., Jones T.R., Lamprecht M.R., Clarke C., Kang I.H., Friman O., Guertin D.A., Chang J.H., Lindquist R.A., Moffat J. (2006). CellProfiler: Image analysis software for identifying and quantifying cell phenotypes. Genome Biol..

[126] Goode A., Gilbert B., Harkes J., Jukic D., Satyanarayanan M. (2013). OpenSlide: A vendor-neutral software foundation for digital pathology. J. Pathol. Inform..

[127] Della Mea V., Baroni G.L., Pilutti D., Di Loreto C. (2017). SlideJ: An ImageJ plugin for automated processing of whole slide images. PLoS ONE.

[128] Marée R., Rollus L., Stévens B., Hoyoux R., Louppe G., Vandaele R., Begon J.-M., Kainz P., Geurts P., Wehenkel L. (2016). Cytomine: An open-source software for collaborative analysis of whole-slide images. Diagn. Pathol..

[129] Bao G., Wang X., Xu R., Loh C., Adeyinka O.D., Pieris D.A., Cherepanoff S., Gracie G., Lee M., McDonald K.L. (2021). PathoFusion: An Open-Source AI Framework for Recognition of Pathomorphological Features and Mapping of Immunohistochemical Data. Cancers.

[130] Sheng V.S., Zhang J. Machine learning with crowdsourcing: A brief summary of the past research and future directions. Proceedings of the AAAI Conference on Artificial Intelligence.

[131] Alaghehbandan R., Perez Montiel D., Luis A.S., Hes O. (2020). Molecular genetics of renal cell tumors: A practical diagnostic approach. Cancers.

[132] Chen R.J., Lu M.Y., Wang J., Williamson D.F., Rodig S.J., Lindeman N.I., Mahmood F. (2020). Pathomic fusion: An integrated framework for fusing histopathology and genomic features for cancer diagnosis and prognosis. IEEE Trans. Med. Imaging.

